# Partial Anomalous Pulmonary Venous Connection Repair: Customized Approach and Outcomes

**DOI:** 10.1007/s00246-021-02583-4

**Published:** 2021-04-02

**Authors:** Lauren Mathis, Danielle Crethers, Bert Buckman, Michael Jensen, Anastasios C Polimenakos

**Affiliations:** 1 Division of Pediatric Cardiothoracic Surgery, Methodist Children’s Hospital Heart Institute, 4410 Medical Drive, Suite 540, San Antonio, TX 78229 USA; 2 Medical College of Georgia, Augusta, GA USA; 3 School of Medical Illustration, Augusta University, Augusta, GA USA

**Keywords:** Sinus venosus ASD, Partial anomalous pulmonary venous connection, Outcomes

## Abstract

Alternative options for the correction of partial anomalous pulmonary venous connection (PAPVC) have been proposed. Each can be associated with variable risk for dysrhythmias, caval or pulmonary venous (PV) obstruction. A selective customized strategy to address PAPVC taking into account atrial shunt (AS) and growth potential was pursued. Between September 2014 and August 2018 21 PAPVC patients were identified. Two levels of reference determined the chosen repair strategy; azygous vein (AzV) and cavoatrial junction (CAJ). Six (Group-A) with PAPVC entering SVC cephalad to AV underwent a combined in-situ cavoatrial autologous reconstruction with atrial appendage advancement flap (CARAF). PAPVC entering caudally to AzV (Group-B) underwent alternative repair (caval division/Warden-type or intraatrial rerouting) (*n* = 15). Age was 8.3 (IQR:4.2–18.5) years for Group-A (vs 11.9; IQR:8.8–34.7 in Group-B) (*p* = 0.07). In Group-A 5(83%) had AS (vs 12[80%] Group-B; *p* = 0.9). None had left SVC in Group-A (vs 1 in Group-B; *p* = 0.9). Preoperative advanced imaging and echocardiographic hemodynamic evaluation was undertaken. Follow-up was complete (median 2.9; IQR:1.2–3.7 years). Freedom from atrial dysrhythmias, caval or PV obstruction was assessed. There were no early or late deaths. ICU and hospital length of stay were 1.8 ± 1.1 and 3.2 ± 0.5 days, respectively. No atrial dysrhythmias occurred postoperatively in Group-A (vs 1 in Group-B; *p* = 0.9). No permanent pacemaker was implanted. All patients remained in normal sinus rhythm. There were no early or late caval/PV obstruction. A customized approach reserves the advantages of each technique tailored to patient’s needs. Expanding surgical capacity with favorable outlook for all PAPVC variations, irrespective of association with AS, can maximize efficiency and reproducibility paired with the lowest morbidity.

## Introduction

Sinus venosus atrial septal defect (SVASD) accounts for 4 to 11% of atrial septal defects (ASD) [1]. It was suggested [2] that superior SVASD originates from failure of atrial wall in-folding between the superior vena cava (SVC) and right pulmonary veins (PV), commonly the upper and middle veins, resulting in un-roofing of the right PVs allowing them to drain into a variable level of SVC or right atrium(RA) [3]. When partial anomalous pulmonary venous connection (PAPVC) is present, most commonly, the anomalous PV is connected to the SVC near the cavoatrial junction (CAJ) or to RA [4]. Although commonly associated with an ASD, 18% of PAPVC cases present with an intact atrial septum [5].

Key elements of any type of PAPVC repair, with or without ASD, should target [4] closure of the inter-atrial communication to create unobstructed drainage of the anomalous PV channel to the left atrium (LA), through a native or surgically created ASD, and of the SVC to the RA. Various techniques [1, 2, 6–11] have been proposed for the surgical re-routing of the neo-PV channel into the LA that might involve caval division. Stenosis of the systemic or neo-PV channels, residual shunting, sino-atrial node (SN) conduction disturbance or supraventricular arrhythmias have been reported [11–13]. Caval division strategy [14] and its modifications were designed to decrease the incidence of systemic and PV complications, as well as, SN dysfunction. One of the challenges, when the caval division is advocated, involves a very selective group of PAPVC where the anomalous PV(s) enter(s) the SVC cephalad to the azygous vein and adjacent to the junction with innominate vein(CIVJ).

Our objective was to review the early and mid-term functional and hemodynamic outcomes with reference to a technical modification, which combines in-situ cavoatrial autologous reconstruction and atrial appendage advancement flap (CARAF) after SVC division, targeting to reserve the benefits of the Warden operation while allowing a tension-free caval-RA anastomosis.

## Methods

**Fig. 1:**
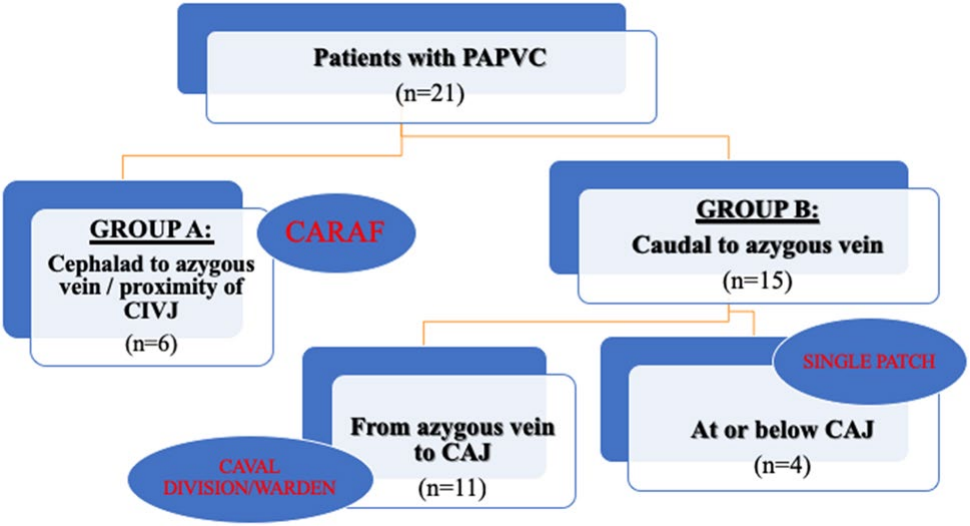
Indications and customized strategy.* CAJ* cavoatrial junction,* CARAF* cavoatrial autologous reconstruction and atrial appendage advancement flap,* CIVJ* cavoinnominate venous junction,* PAPVC* partial anomalous pulmonary venous connection

Between September 2014 and August 2018 21 consecutive patients with PAPVC were identified. PAPVC repair was the index procedure. Patients with associated congenital heart disease where PAPVC was not repaired at the time of the primary operation were excluded from the study. Six patients(Group-A) were associated with PV connection to SVC cephalad to azygous vein, in proximity to the CIVJ, and underwent CARAF. Group-A was compared to patients with anomalous PV entering SVC caudally to the azygous vein(Group-B) who underwent alternative repair strategy (caval division-Warden-type or intraatrial rerouting operation with single patch) (*n* = 15). Following a selective approach our Institution favored CARAF for any PAPVC associated with PV connection to SVC cephalad to the azygous vein, in proximity to the CIVJ (Fig. 1). For PV(s) entering SVC caudally to the azygous vein or within the CAJ, alternative repair strategy was advocated. Our group has not pursued the use of 2-patch approach for any PAPVC type. Clinical data were obtained from a retrospective review of the medical records, operative, echocardiographic, and imaging reports.

The study was approved by the institutional review board. Need for consent was waived.

### Patient Clinical Data

Demographic data included gender, age, weight, associated congenital anomalies and chromosomal anomalies.

Intraoperative data included total cardio-pulmonary bypass(CPB) time, concurrent procedures and arrhythmias.

Postoperative data included the ICU and hospital length of stay(LOS), any associated morbidity, cardiac arrhythmias or SN dysfunction, SVC or PV obstruction and need for reintervention.

### Echocardiographic and Imaging Data

**Fig. 2:**
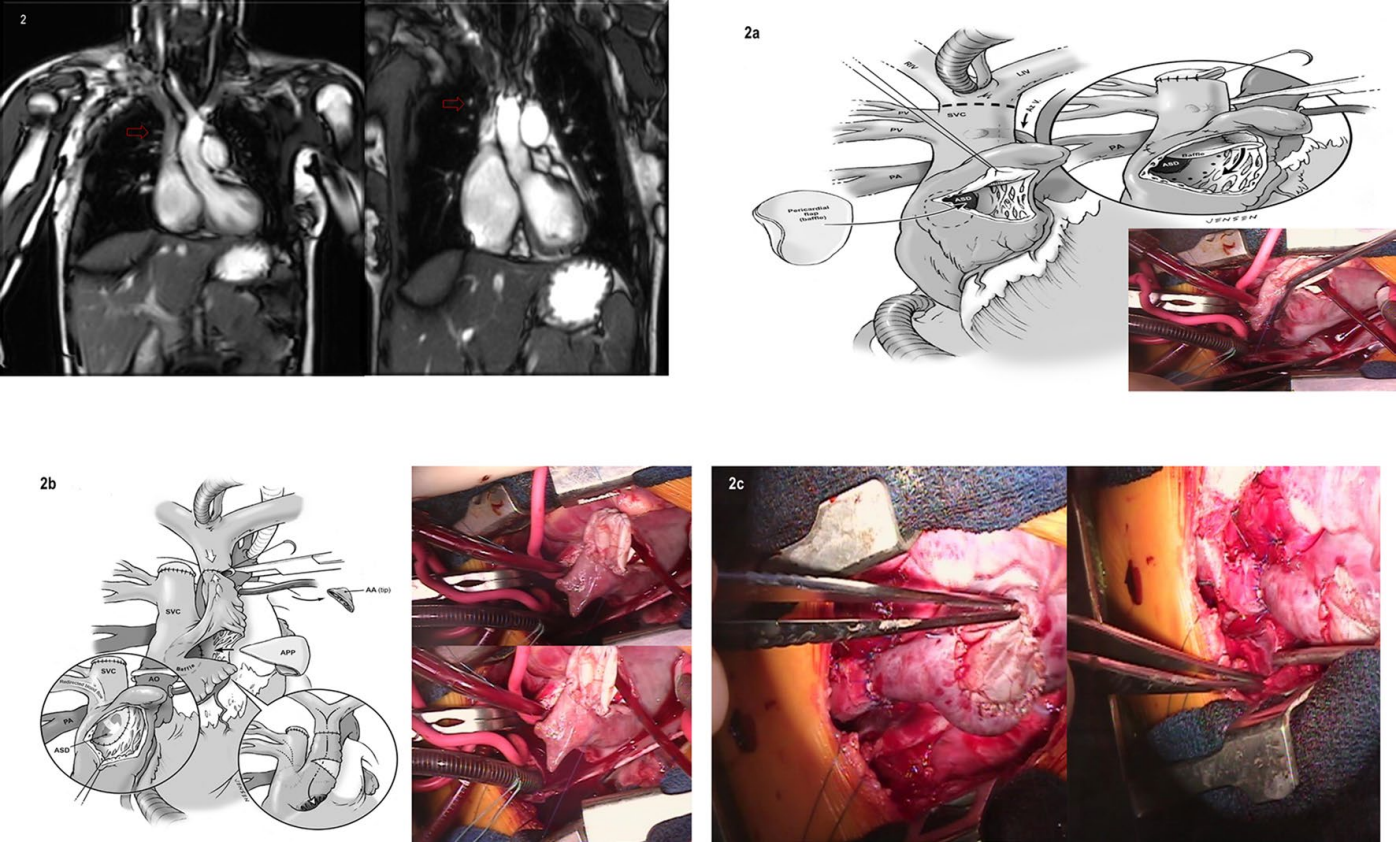
Advanced imaging (MR angiography) and repair strategy. (arrow: indicates the upper anomalous pulmonary venous anatomy and location referenced to the azygous vein). **a** Pulmonary venous channel was baffled with an oversized domed ovale-shaped autologous pericardium patch through the atrial septal defect into the left atrium; **b** A diamond-shaped autologous pericardium was used to allow a cephalad advancement of the right atrial(RA) appendage. The systemic venous channel was reconstituted by suturing the amputated tip of the RA appendage to the cephalad(transected) caval segment. **c** On cardiopulmonary bypass with the heart beating the systemic venous channel is reconstituted by suturing the amputated tip of the RA appendage to the transected caval segment

All patients underwent preoperative advanced imaging (MR, CT Angiogram) to evaluate the pulmonary venous anatomy and plan repair strategy (Fig. 2). Echocardiographic evaluation was performed preoperatively, intraoperatively after CARAF, at hospital discharge and at last follow-up for tricuspid valve(TV) and functional evaluation (2-dimensional doppler and 3-dimensional imaging) of blood flow pattern in systemic and neo-PV channels.

### Surgical Management

A standard median sternotomy is performed. The entrance level of the anomalous PVs into the SVC is adequately visualized after opening the right pleura. The pericardium is opened and the heart exposed. Piece of autologous pericardium is harvested for channeling the anomalous PV to the LA via the ASD and for the atrial wall reconstruction after the RA appendage advancement flap created. The SVC is dissected out to the innominate vein, the innominate vein mobilized to its mid-length and the anomalous PVs are adequately exposed.

CPB is established using ascending aortic and bi- or tri-venous cannulation. The SVC cannula is placed as high as possible in the SVC or in the innominate vein. A persistent left SVC is managed by an additional venous cannula. Normothermia is used in all patients unless otherwise indicated. The azygous vein is divided for complete mobilization of SVC.

Standard Warden operation was performed as previously described [14]. When CARAF was pursued the procedure was staged in 2 components:

1. The channeling of the anomalous PVs via the SVC-RA into the LA under arrested heart
2. The cavo-RA appendage anastomosis on CPB with the heart beating

After cold blood cardioplegic cardiac arrest SVC/innominate and IVC cannulae are snared and a transverse hockey-stick incision is performed extending from the anterior wall of RA appendage base towards the free wall of RA. Through the incision the exposure for the channeling of the anomalous PVs to the LA is excellent without any need for an oblique extension of the incision into the free atrial wall. The right SVC is snared and divided above the insertion of the highest anomalous PV with patching (using autologous pericardium) of its caudal end. Using the SVC-RA native channel the anomalous PVs are redirected away from systemic venous circulation. This is accomplished via the existing superior ASD. If an intact atrial septum is encountered, the fossa ovalis is widely excised and its superior rim resected by extending the incision cephalad and adjacent to the CAJ. The goal is to ensure an unobstructed pathway for the neo-PV channel. The PVs are baffled with an oversized domed ovale-shaped autologous pericardium patch through the ASD into the LA. The SN artery is preserved. To avoid inadvertent injury or SN distortion, suturing of the patch must be exercised with particular attention to avoid full-thickness bites. It is important to suture the patch to the lower rim of the ASD and deviate the suture margin away encroaching onto the lumen of SVC (Fig. 2a/ inset).

Though the transverse atrial incision the tip of RA appendage is conservatively amputated (RA appendage tissue has tremendous pliability). Complete excision of the trabeculations within the RA appendage enhances its expandability and reduces the chance of subsequent adherence or thrombus formation.

For the construction of RA appendage advancement flap a diamond-shaped autologous pericardium is used to allow a cephalad advancement of the RA appendage. The patch is sutured with double-armed polypropylene suture to augment the RA transverse hockey-stick incision (Fig. 2b/inset). The goal is to allow a tension-free in-situ anastomosis of the RA appendage truncated tip with the cephalad (transected) SVC segment without interposition patch or graft. Upon completion of RA appendage advancement flap a RA vent is placed to facilitate a bloodless field during the SVC-RA appendage anastomosis on beating heart.

After de-airing of the heart and cross-clamp removal the remaining of the procedure is carried on CPB with the heart beating. The systemic venous channel is reconstituted by suturing the amputated tip of the RA appendage to the transected SVC segment (Fig. 2c). The suture-line is constructed to reserve growth potential by avoiding purse-string effect. Using polyglyconate monofilament absorbable suture the posterior wall anastomosis is constructed with continuous running fashion. Interrupted technique with polypropylene suture is reserved for the anterior wall anastomosis.

### Follow-Up Data

Follow-up was complete in all patients and spared from era effect. Follow-up data were collected from subsequent clinic visits.

Post-hospital discharge and last follow-up ECG, Holter and echocardiograms were available for all patients. Early and mid-term freedom from conduction system disturbance and supraventricular tachyarrhytmias, SVC or PV obstruction, TV function, need for reintervention was assessed.

### Statistical Analysis

All discrete variables were summarized as percentages and the continuous variables were summarized as mean ± standard deviation or median with interquartile range [25–75 interquartile range (IQR_25-75_)].

Continuous variables were compared by using Mann-Whitney and student-t tests, as appropriate. Fisher’s exact-test and chi-square analyses were used for dichotomous and categorical variables. Univariate analysis was used. All tests of significance were 2-tailed, with P< 0.05 assumed to indicate statistical significance. The SPSS version 22 was utilized to derive the results from the data.

## Results

### Patient Characteristics

Median age was 8.3(IQR, 2.2–18.5) years for Group-A (vs 11.9; IQR, 8.8–34.7 in Group-B) (*p* = 0.07). Mean body weight was 22.5 ± 14.8 kg for Group-A (vs 35.7 ± 13.7 kg) (*p* = 0.05). Ten(48%) were male and 11(52%) were female(Table 1).

**Table 1:** Patient and operative characteristics

Variables	Group A (*n* = 6)	Group B (*N* = 15)	*P* value
*Preoperative variables*			
Age (years; median, IQR)	8.3 (2.2-18.5)	11.9 (8.8-34.7)	0.07
Males/Females	3/3	7/8	
Weight at surgery (kg; mean, SD)	22.5 ± 14.8	35.7 ± 13.7	0.05
Type of atrial shunt			0.01
A. With	5	12	
1. SVASD	*4*	*10*	
2. Ostium secundum ASD	*0*	*1*	
3. PFO	*1*	*1*	
B. Without	1	3	
Concomitant cardiac anomalies			
1. VSD	1	0	0.8
2. Pulmonary valve stenosis	0	1	0.9
3. Left SVC	0	1	0.9
4. TV insufficiency (>mild)	1	2	0.8
No. of anomalous pulmonary veins			
1	2	4	0.8
2	4	10	0.8
3	0	1	0.9
Location of anomalous pulmonary veins			
1. Above azygous vein	6	0	
2. Between azygous vein and atriocaval junction	0	11	
3. At or below atriocaval junction	0	4	
Presenting diagnosis			
1. Murmur/asymptomatic	5	9	0.1
2. Symptomatic	1	6	0.1
Imaging modality for establishing diagnosis			
1. TTE	6	15	
2. MRA	3	9	
3. CTA	2	4	
4. Cardiac cath/angiogram	1	2	
Associated extracardiac / chromosomal abnormalities	1	3	0.7
*Intraoperative variables*			
CBP (min; mean, SD)	51.3 ± 18.5	54.9 ± 14.8	0.8
Cross Clamp (min; mean, SD)	41.9 ± 10.5	46.1 ± 17.5	0.1
Type of repair			
Caval division (Warden or modified)	0	11	
CARAF	6	0	
Single-patch	0	4	
Total additional surgical procedures			
Left SVC intervention	0	1^¶^	0.9
ASD creation (enlargement)	2(1)^§^	5(2)^§^	0.9
TV repair	1	1	0.8
VSD repair	1	0	0.9
Pulmonary valvotomy	0	1	0.9
Intraoperative dysrhythmias	0	1	0.9

*CPB* cardiopulmonary bypass, *PFO* patent foramen ovale, *SVASD* sinus venosus atrial septal defect, *SVC* superior vena cava, *TV* tricuspid valve, *VSD* ventricular septal defect

^§^PFO enlargement

^¶^Unroofed coronary sinus repair

**Fig. 3:**
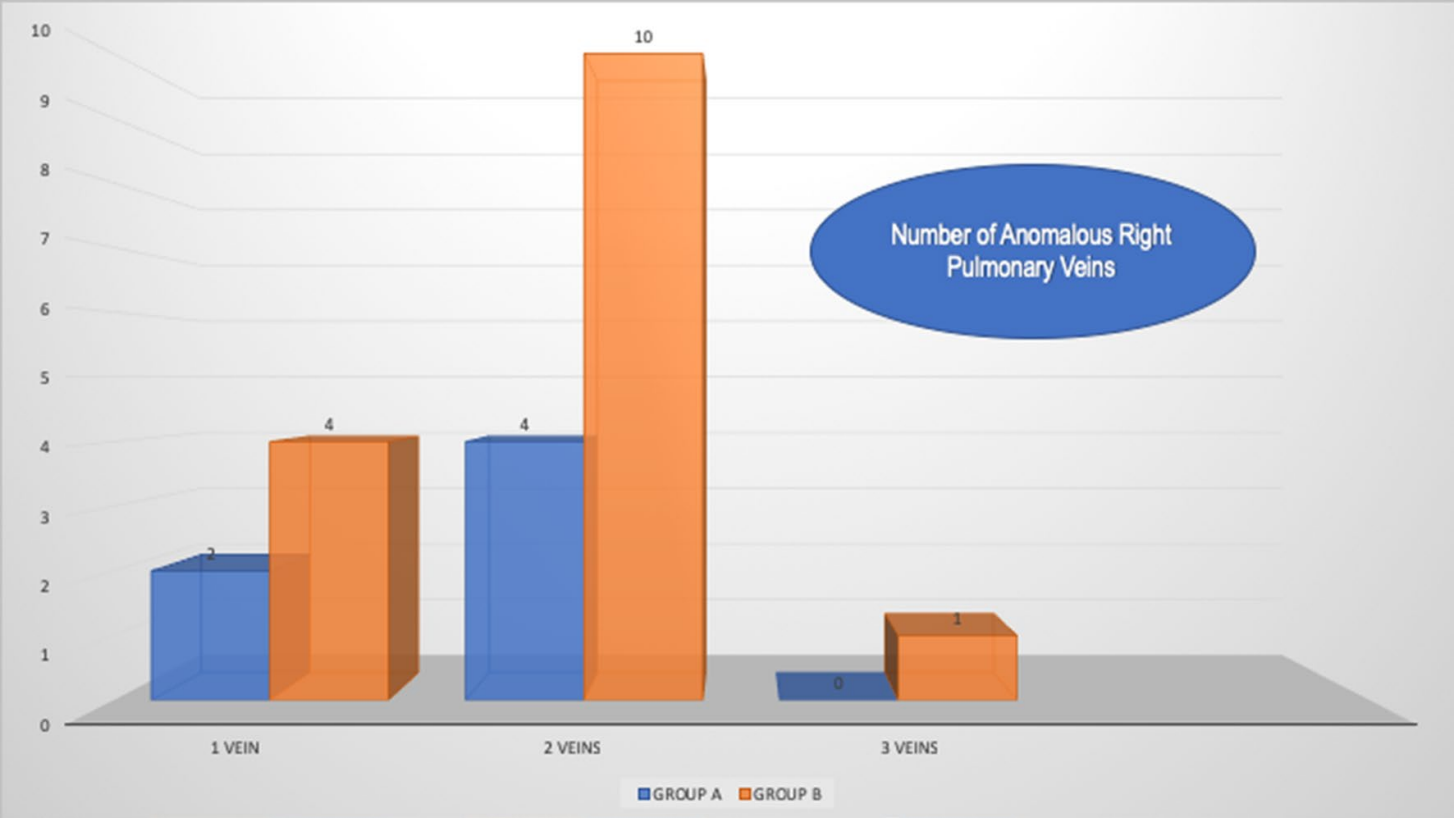
Number of anomalous pulmonary veins involved

In Group-A 4(67%) had an ASD (vs 12[80%] in Group-B). None had left SVC in Group-A (vs 1 without bridging vein in Group-B; *p* = 0.9). The most common anomalous PV drainage combination in Group-A was the right upper and middle lobe PVs (4 patients; 67%). In 4 (of 6) all segments of upper lobe PVs enter SVC above azygous vein. Between the 2 groups most had two anomalous PVs(67%) (Fig. 3). None had an associated left PAPVR. Most common SVASD type was the superior (*n* = 13). Greater then mild TV insufficiency was present is 3(14%). Most common initial clinical presentation was murmur in 14 patients (67%).

Patient characteristics, anatomical details and concomitant diagnoses are depicted in Table 1.

### Early Outcomes

The majority of the patients in Group-B underwent caval division/Warden-type repair (*n* = 9); 6 underwent single-patch repair. The most common associated procedures, other than ASD, was TV repair (*n* = 2; 9.5%). Both patients had RA enlargement with moderate TV insufficiency and were older than 18 years old at the time of the index repair. Specific procedural characteristics and additional procedures at the time of primary PAPVR repair are shown in Table 1.

All patients were extubated in the operating room. There were no early deaths or reoperations. ICU and hospital LOS were 1.8 ± 1.1 and 3.2 ± 0.5 days, respectively, with no difference between groups (*p* = 0.9). No supraventricular dysrhythmias or SN dysfunction occurred during the perioperative period in Group-A compared to 1 in Group-B (p 0.9) (Table 2). One had mild postoperative TV insufficiency. One had postpericardiotomy syndrome that was managed with non-steroidal anti-inflammatory therapy. No evidence of systemic or PV obstruction, residual atrial shunt, or need for reoperations emerged (Table 2).

**Table 2:** Outcomes and follow-up

Variables	Group A (*n* = 6)	Group B (*N* = 15)	*P* value
*Postoperative variables*			
ICU stay (days; mean, SD)	1.7 ± 1.2	1.9 ± 1.3	0.9
Hospital stay (days; mean, SD)	3.1 ± 0.6	3.2 ± 0.4	0.9
Post-cardiotomy syndrome	1	0	0.8
Complications; other	0	1	0.9
Dysrhythmias	0	1	0.9
Use of temporary or permanent pacemaker^¶^	0	0	–
Sinus rhythm^¶^	6	15	–
Systemic venous stenosis/obstruction^¶^	0	0	–
Pulmonary venous stenosis/obstruction^¶^	0	0	–
TV insufficiency (>mild)^¶^	0	1	0.9
*Follow-up variables*			
30-day readmission	0	0	
Permanent pacemaker^§^	0	0	
Dysrhythmias^§^	0	0	
Normal sinus rhythm^§^	6	15	
Systemic venous stenosis/obstruction^§^	0	0	
Pulmonary venous stenosis/obstruction^§^	0	0	
TV insufficiency^§^	0	1	
Reintervention(any)	0	0	
NYHA class 1 (only in adults)	2 (of 2)	0 (of 9)	

*TV* tricuspid valve

^¶^At hospital discharge

^§^At last follow up

### Mid-Term Outcomes

Median follow-up was 2.9 (IQR 1.2–3.7) years and synchronous between the 2 groups. No 30-day readmissions occurred. There were no late deaths.

**Fig. 4:**
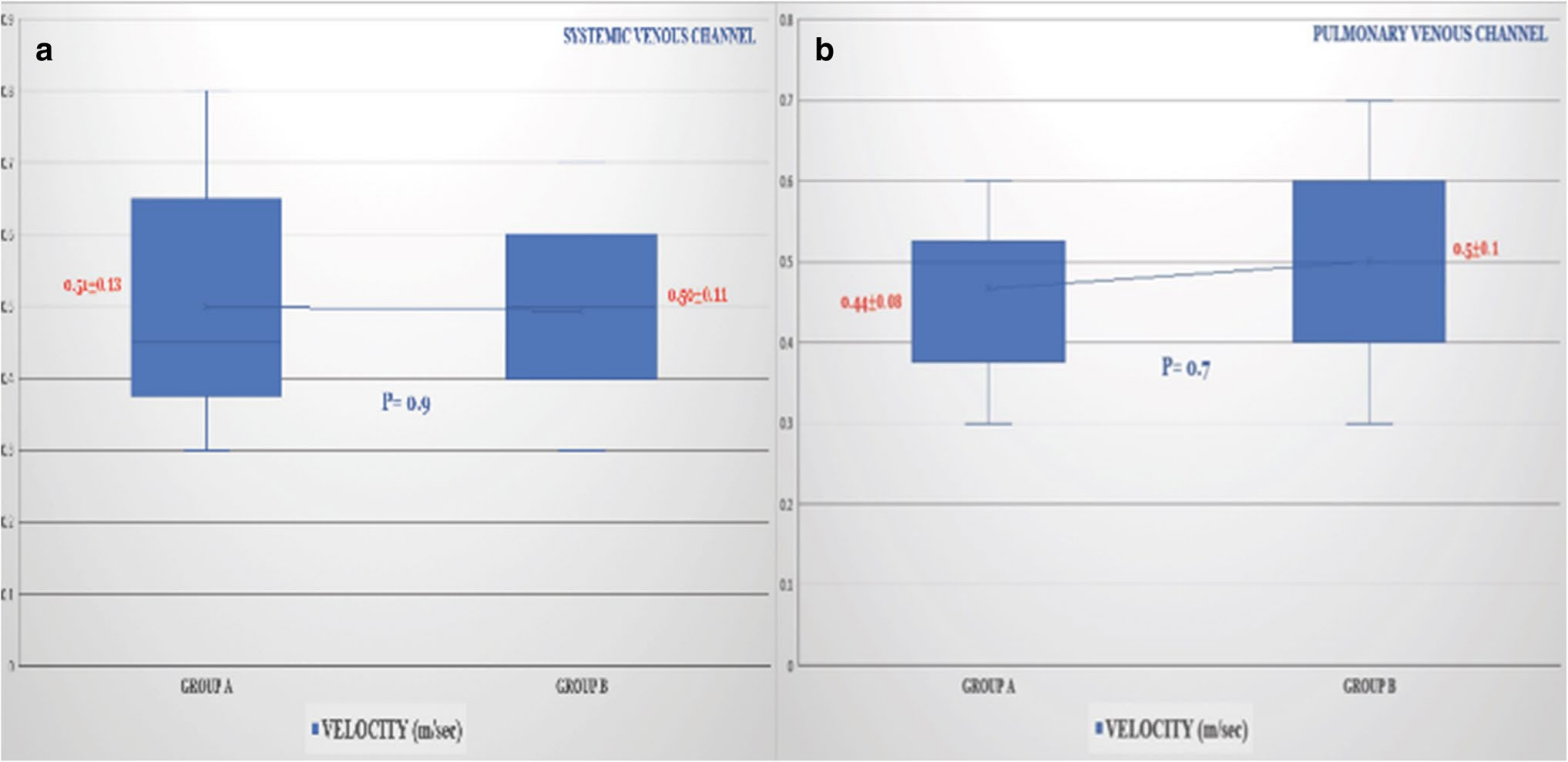
Velocity by echocardiographic doppler assessment of **a **systemic and **b **pulmonary venous channels

All patients (both groups) had no evidence of systemic or PV obstruction or need for reoperations at last follow-up (Table 2). Specifically, by echocardiography, systemic and neo-PV channels flow velocity in Group-A was 0.51 ± 0.13 and 0.44 ± 0.08 m/s (vs 0.5 ± 0.1 and 0.5 ± 0.11 from Group-B), respectively. Two-dimensional Doppler and 3-dimensional echocardiography showed widely unrestricted anastomoses, unobstructed, non-turbulent systemic and PV flow (Fig. 4a,b).

All patients maintained normal sinus rhythm(NSR) with no supraventricular dysrhythmias at last follow-up. No permanent pacemaker system was implanted. One with mild TV insufficiency at hospital discharge had no worsening of valve function. Adult patients remained asymptomatic with excellent functional status (NYHA Class-1) (Table 2).

## Discussion

As literature indicates, SVASD, which is commonly located at the SVC-RA junction, accounts for 10% of all ASDs with 90% being associated with PAPVC [15, 16]. Various repair techniques have been proposed to accommodate PAPVC. For pediatric patients, maintaining growth potential is off the essence. The location of SN, SN artery, crista terminalis and the diameter of the SVC are critical considerations [2]. Conventional procedures included single [1, 11, 17] or two-patch [18–20] approaches with incisions across the cavo-atrial junction, alternative atrial appendage flap techniques [21] and caval division with reconstitution of flow to RA via RA appendage with or without cavoatrial augmentation [14, 21].

Results have generally been favorable, although clinically significant stenoses of the systemic or PV channels (up to 27% in selected studies) and dysrhythmias (up to 55%) have been reported [8, 17–20, 22, 23]. While concerning [17, 24], complications related to systemic or PV channel integrity and SN function or SVA have been mitigated with encouraging outcomes referenced to Warden operation and modifications [13]. Said et al. [25] compared 3 repair strategies. In the single-patch postoperative supraventricular arrhythmias, SVC and PV obstruction were 8%, 5%, and 5%, respectively, compared to 12%, 4%, and 12%, respectively, in the double-patch group, and 22%, 7%, and 0, respectively, in the caval division/Warden-type repair.

We applied a selective customized strategy to address PAPVC entering SVC at different levels, taking into account the association with atrial shunt and growth potential. Our approach calls for utilizing most suitable repair, paired with the lowest possible early and late morbidity, for maximizing efficiency and reproducibility. A customized approach reserves the advantages of each isolated technique tailored to the needs of each patient. Thus, a single repair approach cannot fit all. We advocate that 2 levels of reference can determine the chosen repair strategy; namely azygous vein and CAJ. Specifically, CARAF is advocated for those with PAPVC entering SVC cephalad to azygous vein. When PAPVC enters SVC caudally to azygous vein, management can be directed based on a second level of reference; the CAJ. If PAPVC (if more than one veins) enter SVC above CAJ, then caval division/Warden-type repair shall be favored. Alternatively, if PAPVC enters at or below the CAJ, then, the likely course of action calls for intra-atrial rerouting operation with single patch. For patients with characteristics demonstrated in Group-A CARAF is an improvement over previously reported approaches for the following reasons: (1) CAJ preservation and SN integrity; (2) A tension-free cavo-atrial connection with growth potential (with the use of the RA appendage as an advancement flap) particularly important for children and adolescents as most of our patients were; (3) Construction of cavo-atrial connection on beating heart CPB, thereby allowing for proper orientation and tailoring which can decrease the likelihood for distortion or narrowing of neo-caval channel; (4) Expanding surgical capacity with favorable outlook for all PAPVC variations, irrespective of the level of anomalous PVs entering SVC and its association with atrial shunt.

One important observation is that our CARAF group had younger patients. This may be due to referral patterns. There was no age effect on early or late development of atrial dysrhythmias, systemic or PV channel stenoses. As scarcely reported [26], older age might be associated with more pronounced adverse cardiac events. An association between older age and atrial tachyarrhythmias needs to be further evaluated by future prospective multisite studies to guide management. Our study reiterates the fact that PAPVC, irrespective of atrial shunt, can be effectively repaired at an earlier age or soon after the initial diagnosis using a customized approach with tailored repair strategy.

As others reported [11, 23] caval division strategies have mitigated incidence of SN dysfunction and dysrhythmias. All patients in our series remained in NSR at last follow-up with no need for pacemaker. This is consistent with others experience when 1-patch or 2-patch intraatrial rerouting techniques were used [20]. This is an inherent risk for any repair strategy where incisions are directed towards or in the proximity of SN. Due to the variable anatomical course of SN artery, the risk of SN dysfunction, even in the absence of injury of the node itself, cannot be undermined. None of the patients in this cohort developed mid-term supraventricular tachyarrhythnias. Long-term follow-up data will determine if this trend continues.

One of the highlights of our customized approach is that use of CARAF effectively prevents early or mid-term caval stenoses. The primary advantage of this procedure is that a right atrial incision extending across the CAJ or near the SN is not necessary. In addition, the cavoatrial anastomosis does not involve interposition patch or graft allowing for growth potential. Interposition graft in accomplishing a tension-free cavoatrial anastomosis in older patients has been advocated by some [27]. Our group supports the concept of a strategy which promotes anastomotic growth potential, particularly in the younger patients. As demonstrated by echocardiographic hemodynamic assessment, using 2-dimensional Doppler and 3-dimensional echocardiography widely unobstructed, non-turbulent systemic flow was clearly shown.

As noted by others [28] following Warden-type repair risk for PV obstruction is limited and this repair approach is favorably suited for younger patients with PAPVC entering the SVC. In our series no patients, who underwent a caval division procedure or CARAF in both groups, had any early or late PV obstruction and flow, as demonstrated by echocardiographic hemodynamic assessment, remains non-turbulent and widely unobstructed.

It is reported [29] that caval division with cavoatrial anastomosis might be small or under tension and, thus, carries the disadvantage of acute thrombosis or subsequent stenosis. CARAF provides a reproducible and reliable strategy for these particular cases, especially when treated earlier in life. The growth potential of both venous channels is effectively preserved. Furthermore, postoperative anticoagulation therapy was spared. Recent report [30] described an approach using double-decker strategy. Authors highlighted that using this approach atriotomy is limited within a relatively safe zone from the SN (and artery) and avoids the end-to-end cavoatrial anastomosis. They advocated that systemic venous chamber integrity is preserved by assuring no histological discontinuity at its posterior wall. Our group has no experience with this approach.

Echocardiographic assessment was performed in all patients to assess the systemic and PV channel, as well as, TV functional capacity. Specifically, cardiac echocardiography velocity measurements, although operator-dependent and limited in their ability to calculate blood flow volume and measure wall shear stress, can provide meaningful functional evaluation of blood flow velocity and pattern of neo-systemic and neo-PV channels. Our results indicated that no early or late PV and SVC flow disturbance was recorded. In addition, wide pathway of the intra-atrial baffle was clearly shown. Recently, others [30, 31] have advocated the value of 4D-flow MRI as a novel blood flow imaging technology in vivo for systematic evaluation of hemodynamics, the actual measured values for flow streamline (blood flow combined with flow velocity) and wall shear stress. Our study does not provide insight related to this imaging modality, but our program is to incorporate this type of assessment for all patients with anomalous PV connection (partial or total). Echocardiography following TV repair determined no deterioration of TV functional capacity at last follow-up. The small sample and limited long-term data precluded meaningful interpretation of valve, systemic and PV channels integrity associated with PAPVC repair and its impact.

There are several limitations to this study. It is single-site retrospective study subject to selection bias and lack of randomization. There was no historical control group for comparison. The small sample size made rather difficult to draw clinically significant albeit statistically significant, conclusions from subgroup analysis. SN disturbance, occurrence of long-term atrial dysrhythmias or fate of neo-systemic and neo-PV channels patency may be underestimated. Along the same line, inferences regarding the impact of the operative technique employed to future cardiac events would not be scientifically sound.

In conclusion, surgical repair of PAPVC using CARAF technique appears to be effective and reproducible with no associated morbidity in cases where conventional strategies might carry risk for SN disturbance and caval or PV channel obstruction. Customized approach can be clearly advocated. CARAF for treatment of this subset of PAPVC is associated with low mortality and morbidity, favorable long-term and hemodynamic outcomes related to caval or PV channels. Growth potential is effectively preserved. Large multi-site studies might help determine future guidelines in pursuing a targeted strategy tailored to this subset of patients.

## Abbreviations

CAj: Cavoatrial junction
CARAF: Cavoatrial autologous reconstruction and atrial appendage advancement flap
CIVJ: Cavoinnominate venous junction
CPB: Cardiopulmonary bypass
LA: Left atrium
LOS: Length of stay
NSR: Normal sinus rhythm
PAPVC: Partial anomalous pulmonary venous connection
PV: Pulmonary vein
RA: Right atrium
SN: Sino-atrial node
SVASD: Sinus venosus atrial septal defect
SVC: Superior vena cava
TV: Tricuspid valve
